# Clinical and research updates on the VISTA immune checkpoint: immuno-oncology themes and highlights

**DOI:** 10.3389/fonc.2023.1225081

**Published:** 2023-09-15

**Authors:** Randolph J. Noelle, J. Louise Lines, Lionel D. Lewis, Robert E. Martell, Thierry Guillaudeux, Sam W. Lee, Kathleen M. Mahoney, Matthew D. Vesely, Jerome Boyd-Kirkup, Dhanya K. Nambiar, Andrew M. Scott

**Affiliations:** ^1^ ImmuNext Inc., Lebanon, NH, United States; ^2^ Department of Microbiology and Immunology, Geisel School of Medicine at Dartmouth, Hanover, NH, United States; ^3^ Department of Microbiology and Immunology, Dartmouth Cancer Center, Geisel School of Medicine at Dartmouth, Hanover, NH, United States; ^4^ Section of Clinical Pharmacology, Department of Medicine, Geisel School of Medicine at Dartmouth and Dartmouth Cancer Center, Hanover, NH, United States; ^5^ Curis, Inc., Lexington, MA, United States; ^6^ Division of Hematology/Oncology, Tufts Medical Center, Boston, MA, United States; ^7^ Kineta Inc., Seattle, WA, United States; ^8^ Yale University School of Medicine, New Haven, CT, United States; ^9^ Department of Medical, Division of Medical Oncology, Beth Israel Deaconess Medical Center, Harvard Medical School, Boston, MA, United States; ^10^ Department of Medical Oncology, Dana-Farber Cancer Institute, Harvard Medical School, Boston, MA, United States; ^11^ Department of Dermatology, Yale School of Medicine, New Haven, CT, United States; ^12^ Hummingbird Bioscience, Houston, TX, United States; ^13^ Department of Radiation Oncology, Stanford School of Medicine, Stanford, CA, United States; ^14^ Olivia Newton-John Cancer Research Institute and School of Cancer Medicine, La Trobe University, Melbourne, VIC, Australia; ^15^ Department of Molecular Imaging and Therapy, Austin Health and Faculty of Medicine, University of Melbourne, Melbourne, VIC, Australia

**Keywords:** VISTA, checkpoint inhibitors (ICIs), immunotherapy, PD-1, myeloid, immune checkpoint, immuno-oncology (IO)

## Abstract

Immune checkpoints limit the activation of the immune system and serve an important homeostatic function but can also restrict immune responses against tumors. Inhibition of specific immune checkpoint proteins such as the B7:CD28 family members programmed cell death protein-1 (PD-1) and cytotoxic T-lymphocyte antigen-4 (CTLA-4) has transformed the treatment of various cancers by promoting the anti-tumor activation of immune cells. In contrast to these effects, the V-domain immunoglobulin suppressor of T-cell activation (VISTA) regulates the steady state of the resting immune system and promotes homeostasis by mechanisms distinct from PD-1 and CTLA-4. The effects of VISTA blockade have been shown to include a decrease in myeloid suppression coupled with proinflammatory changes by mechanisms that are separate and distinct from other immune checkpoint proteins; in some preclinical studies these immune effects appear synergistic. Given the potential benefits of VISTA blockade in the context of cancer therapy, the second Annual VISTA Symposium was convened virtually on September 23, 2022, to review new research from investigators and immuno-oncology experts. Discussions in the meeting extended the knowledge of VISTA biology and the effects of VISTA inhibition, particularly on cells of the myeloid lineage and resting T cells, as three candidate anti-VISTA antibodies are in, or nearing, clinical development.

## Introduction to the VISTA immune checkpoint

The landscape of cancer management has been transformed enormously by advances in our understanding of immune system checkpoints and the blockade of their downstream signaling pathways. Treatment of appropriate malignancies with immune checkpoint inhibitors (ICIs) targeting cytotoxic T-lymphocyte antigen-4 (CTLA-4) and programmed cell death protein-1 (PD-1, along with its ligand, PD-L1) has markedly improved outcomes for patients with tumors by promoting activation of the adaptive immune system. In contrast, a related immune checkpoint protein, V-domain immunoglobulin (Ig) suppressor of T-cell activation (VISTA), serves in a homeostatic role to regulate the steady state of both the lymphoid and myeloid lineages of the resting immune system (1). VISTA is also known as PD-1H, c10orf54, VSIR, SISP1, B7-H5, DD1a, Gi24, and Dies1 (2, 3).

VISTA demonstrates similarity to the B7 family of ligands – sharing 25% protein sequence identity with PD-L1 – and receptors such as the CD28 family, but has unique structural features, expression patterns, and functions (4, 5). The expression of VISTA on tumor or immune cells may significantly affect tumor growth and antitumor immunity, with VISTA acting as a receptor, a ligand, or both (1, 6). Early studies by Chen and colleagues identified VISTA as a negative regulatory receptor on T cells (2, 7, 8). Ligands that have been identified as potential binding partners for VISTA include PSGL-1 (at low pH), VSIG-3, Galectin-9, LRIG1, and Syndecan-2 (9–13). Additionally, VISTA has been shown to form homophilic interactions at intercellular junctions of apoptotic cells and macrophages (14). The physiological relevance of these potential interactions remains uncertain at present and requires further investigation (1).

VISTA is expressed on most hematopoietic cells and is highly expressed on many cells in the myeloid lineage (1, 4). Resting T cells also express VISTA, which is transiently downregulated upon activation and then re-expressed on memory T cells (1, 4). VISTA serves as a checkpoint for resting T cells that negatively regulates the earliest stages of T-cell activation and the transition from quiescence to priming, suggesting that the VISTA checkpoint regulates the activation of resting T cells prior to the involvement of the PD-(L)1 and CTLA-4 checkpoints (1, 15).

Given its role in the regulation of the immune system, the expression and functions of VISTA in cancers are currently under active investigation. In preclinical models, high levels of VISTA expression are found on tumor-infiltrating leukocytes and myeloid-derived suppressor cells (MDSCs) (1, 6, 15–17). VISTA expression levels have also been examined on malignant cells. While several tumor types have been reported to express elevated levels of VISTA, careful assessment is still needed to interpret the significance of this finding, given that infiltrating VISTA-expressing immune cells may also be present (1, 6, 15–17). In several tumor types, such as melanoma and ovarian cancer, VISTA expression appears to correlate with disease progression and is indicative of poor survival in some settings (4). Encouragingly, VISTA-targeting agents have demonstrated promising findings in multiple preclinical tumor models (1, 4).

**Figure 1:**
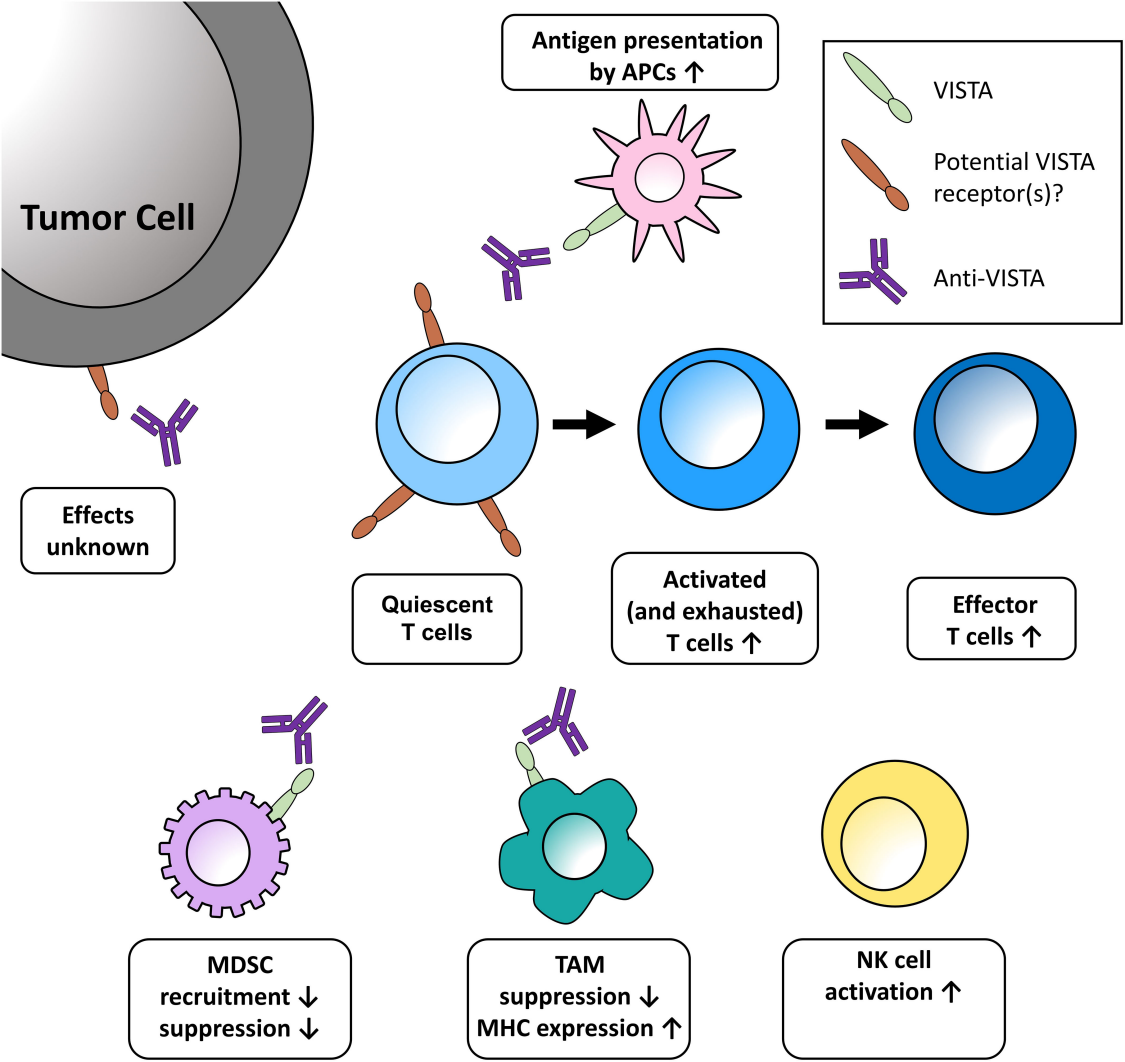
Potential effects of VISTA blockade in the tumor microenvironment discussed at the meeting. One role of VISTA is to serve as a checkpoint on naïve T cells, impeding the earliest stages of T-cell activation. Blockade of VISTA brings T cells out of quiescence, then acts to expand and transform activated and exhausted T cells into effector cells. Further effects of VISTA blockade in the TME include enhanced presentation of antigens on monocytes, decreased recruitment of MDSCs, increased release of T-cell activating factors, and increased maturation of NK cells. These effects are further bolstered by increased MHC expression on TAMs and the reduced expression of genes promoting T-cell quiescence. The functional relevance of VISTA expression on tumor cells has yet to be uncovered. APC, antigen-presenting cell; MDSC, myeloid-derived suppressor cells; MHC, major histocompatibility complex; NK, natural killer; TAM, tumor-associated macrophage.

As an emerging immune checkpoint, VISTA represents a compelling new molecular treatment target under active investigation. Three distinct anti-VISTA antibodies are currently approaching or moving forward into clinical development. For the second consecutive year, a VISTA symposium was held virtually on September 23, 2022, to support efforts by investigators and immuno-oncology experts, accelerate our understanding of VISTA, and promote the development of potential therapeutics targeting this protein. Among the key findings of the second Annual VISTA Symposium was a growing awareness that VISTA inhibition exerts multiple effects on the immune system that are distinct from other ICIs (**Figure 1**). In particular, several presentations outlined the effects of VISTA blockade, including a decrease in myeloid suppression of immune responses coupled with proinflammatory changes in the tumor microenvironment (TME) by mechanisms separate and distinct from PD-1 and CTLA-4 inhibition. New preclinical and clinical data were described for three candidate anti-VISTA antibodies currently in development (CI-8993 [Curis, Inc.], HMBD-002 [Hummingbird Bioscience], and KVA12123 [Kineta, Inc.]).

## VISTA and the suppression of immune responses by myeloid cells

It is now well established that myeloid cell reprogramming by tumors is an important way in which they evade the immune response (18, 19). Research suggests that the VISTA checkpoint plays an important role in the regulation of MDSCs, which inhibit the responses of the immune system in some malignancies. MDSCs exhibit potent immunosuppressive activity and higher numbers of circulating MDSCs are closely linked to poor clinical outcomes in cancer (18). In this way, VISTA shares some functional similarity to leukocyte Ig-like receptor subfamily B ICIs such as LAIR1, which have been demonstrated to support the immunosuppressive activity of myeloid cells (20). A defining characteristic of MDSCs is their interference with the immune responses mediated by T cells, B cells, and natural killer (NK) cells (18). Patients with acute myeloid leukemia have increased expression of VISTA on MDSCs, and VISTA is a critical component of the MDSC-mediated inhibition of CD8+ T-cell responses (21). In murine cancer models, VISTA knockout resulted in reduced migration of macrophages and MDSCs into the TME. In contrast, treatment with an anti-VISTA monoclonal antibody (mAb) resulted in a reduction of tumor-infiltrating MDSCs accompanied by an increase of tumor-infiltrating CD4+ and CD8+ T cells (16, 22). An emerging model hypothesizes that VISTA promotes both migration of inhibitory myeloid cells (such as MDSCs) to the TME and their suppressive effects on the immune system within the TME (1, 4). This is supported by another study using the CT26 colon cancer model that shows the role of VISTA in MDSC-mediated T-cell suppression under hypoxic conditions and indicates that targeting VISTA may counteract the dampening effect of hypoxia on antitumor immunity (23).

Several presentations at the symposium described aspects of VISTA regulation within the myeloid compartment, specifically regarding the activity of MDSCs. Research from Dr. Sam W. Lee’s group at Yale School of Medicine revealed that the *in vitro* inhibition of CD4+ T cells by MDSCs was reversed by treatment with antibodies targeting VISTA and in VISTA^–/–^ syngeneic murine tumor models. A second preclinical murine study presented by Dr. Louise Lines of Dartmouth demonstrated that treatment with an anti-VISTA antibody exerted multiple effects on tumor-associated macrophages (TAMs), including diminished myeloid suppression of T-cell proliferation, increased major histocompatibility complex expression on TAMs, and reduced expression of genes promoting T-cell quiescence. Taken in combination, these findings extend the knowledge of the roles of VISTA in myeloid cells and further support the rationale for targeting VISTA.

## Clinical prospects for VISTA inhibition

High levels of VISTA expression have been reported in patient samples from multiple forms of cancer (**Table 1**) (21, 24–39). However, the conclusions drawn from these patterns of expression are still emerging. For example, in patients with melanoma, any level of VISTA expression in the tumor-infiltrating inflammatory cells of their primary melanoma decreased survival, whereas in renal cell carcinoma (RCC), VISTA-positive immune cells homing to the venous tumor thrombus, but not to the primary RCC itself, indicated poor prognosis (29, 40). Conversely, high levels of VISTA expression have also been linked with improved survival outcomes in some settings, suggesting that further research is required to assess VISTA expression and function within the TME (41).

**Table 1:** Patterns of VISTA expression in different cancers.

Cancer type	Pattern of VISTA expression	Citation
AML (21)	Expression in MDSCs of the peripheral blood, 54.3% AML vs 33.3% in healthy controls (*p*=0.0262)	Wang, 2018
Breast (24–26)	In triple-negative, immune cells 87.8% (223/254) and tumor cells 18.5% (47/254)	Cao, 2021
	Expression in 14.2% of 14,897 cancer cells vs 7.6% of 7,320 normal, adjacent cells	Xie, 2020
	In IDC, expression on 29.1% (267/919) of immune cells and 8.2% (75/919) of tumor cells	Zong, 2020
Colorectal (27, 28)	Expression in normal tissue, para-tumor, and tumor, but with the highest expression in tumors	Xie, 2018
	Expression is significantly higher in MSI+ CRC tumors compared to MSS tumors	Zaravinos, 2019
Melanoma (29)	Expression is associated with significantly worse disease-specific survival by univariate analysis (HR=3.57, *p*=0.005) and multivariate analysis (HR=3.02, *p*=0.02)	Kuklinski, 2018
Mesothelioma (30–32)	Expression is higher in epithelioid type (>78%) than sarcomatoid type (<55%) (*p*<0.0001)	Chung, 2020
	Expression in 85% (270/319) of MPM samples, higher in epithelioid (88%) than sarcomatoid	Muller, 2020
	Expression detected in all MPM cases (n=160), comprising epithelioid (n=101), biphasic (n=38), and sarcomatoid (n=21)	Rooney, 2019
NSCLC (33)	Expression in 99% of 758 stage I–IV samples; higher in stromal cells (98%) than in tumor cells (21%)	Villarroel-Espindola, 2018
Ovarian (34)	Expression in 51.4% (75/146) of samples; 28.8% of tumor cells, 35.6% of immune cells, and 4.1% of endothelial cells	Zong, 2020
Pancreatic (35–37)	Predominantly expressed on CD68+ macrophages in PDAC	Blando, 2019
	Expression detected in IPMN (4/4) and adenocarcinoma (13/15)	Byers, 2015
	Positive staining was detected in 88.5% of 52 human samples	Liu, 2018
Prostate (38)	No expression on CD4/CD8 T cells in pre-treatment tumor tissues, but detected on 4% of CD4 T cells and 7% on CD8 T cells after ipilimumab therapy; the expression on CD68+ macrophages increased ~4-fold (from 7% to 31%)	Gao, 2017
Renal (39)	Higher expression is seen in clear cell RCC tumors than in non-tumoral tissues	Hong, 2019

AML, acute myeloid leukemia; HR, hazard ratio; IDC, invasive ductal carcinoma; IPMN, intraductal papillary mucinous neoplasm; MDSC, myeloid-derived suppressor cells; MPM, malignant pleural mesothelioma; MSI+, microsatellite unstable; MSS, microsatellite stable; NSCLC, non-small cell lung cancer; PDAC, pancreatic ductal adenocarcinoma; RCC, renal cell carcinoma.

New research was presented at the symposium exploring the opportunities for VISTA blockade in potential target pathologies. New data from the laboratory of Dr. Kathleen Mahoney at Dana-Farber Cancer Institute and Beth Israel Deaconess Medical Center revealed a high expression of VISTA in samples of kidney cancer at levels that exceed those of PD-1 and PD-L1. Histology studies of clear cell kidney cancer found VISTA expression on tumor cells in a subset of cases, but the highest VISTA expression was observed on infiltrating immune cells. In the case of head and neck cancer (HNC), Dr. Dhanya Nambiar of Stanford University School of Medicine shared findings demonstrating that radiation therapy contributed to the migration of circulating MDSCs with an enhanced expression of VISTA, and VISTA blockade significantly improved radiation treatment response in mouse models of HNC.

Cutaneous malignancies, including melanoma and cutaneous squamous cell carcinoma (cSCC), were discussed by Dr. Matthew Vesely of Yale University School of Medicine, who described new immunological findings demonstrating frequent detection of VISTA expression in melanoma (~50% of samples), particularly in CD11b myeloid cells, and this specific form of expression was found to be associated with recurrence and poor survival. Consistent with findings from HNC, the levels of VISTA expression were shown to exceed that of PD-L1, and there was a poor correlation between VISTA and PD-L1 expression in the melanoma samples studied. In cSCC, VISTA and PD-L1 were expressed in a subset of samples, again with poor correlation between the two checkpoints and the highest VISTA expression seen in CD11b cells.

## VISTA inhibition promotes a proinflammatory TME by mechanisms distinct from PD-1 and CTLA-4 inhibition

It is believed that the suppressive effects of MDSCs and TAMs can, in some cases, limit the therapeutic impact of T-cell–directed checkpoint therapies such as those targeting PD-1 or CTLA-4. The constitutive expression of VISTA observed in multiple immune cell lineages (particularly myeloid lineages and resting T cells) suggests that this regulatory protein may act as a “rheostat” to actively normalize immune responses by dampening excessive activation at the earliest stages of the immune response (4). This model of VISTA’s function is important compared to other negative checkpoint regulators since CTLA-4 and PD-1 are induced after activation, indicating that the VISTA checkpoint may be a hurdle to overcome to initiate an immune response (4, 17).

Ongoing research in preclinical models has established the role of the VISTA checkpoint in limiting immune responses. In the CT26 model of colorectal cancer, single-agent anti-VISTA treatment of small tumors resulted in slowed tumor growth (42). Similarly, while large CT26 tumors showed complete adaptive resistance to treatment with anti-PD-1/CTLA-4 antibodies, the addition of an anti-VISTA antibody led to the resolution of half the previously resistant tumors (42). Sequencing of tumor-specific CD8+ T cells revealed that anti-VISTA therapy activated T-cell pathways highly distinct from, and complementary to, those activated by anti-PD-1 therapy, with VISTA-targeting therapy reducing the regulators of T-cell quiescence (42). VISTA blockade in tumor models has been reviewed more comprehensively than this paper allows, for example by Huang et al. and Yum et al. (43, 44). Importantly, VISTA blockade has also demonstrated a combinatorial effect combined with CTLA-4 blockade to induce tumor regression in a murine model of SCC (45). These data indicate that anti-VISTA treatment has the potential to overcome adaptive resistance that arises in treatment strategies inhibiting PD-1 and/or CTLA-4.

From a therapeutic perspective, there are potential risks associated with immune system stimulation *via* anti-VISTA therapy (3). These risks could potentially be amplified when combined with other ICIs, such as those targeting PD-1 and CTLA-4, which have already been associated with immune-related adverse events (3, 46). Blocking VISTA may result in decreased peripheral tolerance through T-cell quiescence and thus development of auto-inflammatory conditions (3). Another concern is cytokine release syndrome (CRS), discussed below.

Presentations during this symposium reinforced the premise that inhibiting or removing VISTA increases activation of the immune system in a manner distinct from the most common ICIs currently employed in clinical practice. Dr. Randolph J. Noelle of Dartmouth proposed a conceptual framework that VISTA and CD28 function as mirror images of one another, with CD28 acting to amplify the development of immune responses while VISTA serves to dampen activation and promote quiescence. This model was supported by new research presented from the Noelle laboratory demonstrating that a loss of VISTA activity enhances the development of autoimmunity in mouse models.

Dr. Lines presented findings from a murine model of colon cancer, revealing that anti-VISTA treatment increased immune cell density in the TME. Findings from her laboratory showed increased post-treatment infiltration of NK cells in addition to CD45+, CD8+, and CD4+ T cells relative to the non-responders. Synergy was observed with the addition of anti-PD-1 and anti-CTLA-4 treatments relative to monotherapy, with further reductions in tumor growth and a reduction of the suppressive character of myeloid cells in the TME. The combination of VISTA and PD-1/CTLA-4 blockade was also shown to exert complementary effects on T cells, leading to an increased population of mature cells required for tumor cell killing and supporting the hypothesis that anti-VISTA treatment may ameliorate the adaptive resistance that can develop with contemporary ICI therapies (42).

An area of active investigation is the role of VISTA in T cells. The Noelle group previously demonstrated that the VISTA checkpoint limits the activation of naïve T cells and maintains peripheral tolerance (15). New findings from the Noelle group described an intriguing role for VISTA on both CD4+ and CD8+ memory T cells. Studies of VISTA knockout mice revealed that memory T cells were increasingly proinflammatory in mice that lacked VISTA, with reduced expression of co-inhibitory molecules. These findings raised multiple questions for future study, including the influences of VISTA on the exhaustion and future phenotype of memory T cells and VISTA’s influence on the survival and effector functions of regulatory T cells.

## Antibodies targeting VISTA are currently in development

The promise of targeting the VISTA checkpoint in multiple cancers has motivated the development of mAbs targeting VISTA to block its function, and updates regarding three unique anti-VISTA mAbs that are currently in or about to enter human clinical trials were presented at the symposium.

The first anti-VISTA antibody to be assessed in humans is CI-8993 (Curis, Inc.). CI-8993 has putative binding sites at 4 residues within the C-C’ loop of VISTA, has been demonstrated to block interaction with both with PSGL-1 and VSIG-3 binding, and is constructed with an active IgG1 Fc domain that facilitates interactions with Fc gamma receptors and myeloid cells and supports antibody-dependent cell cytotoxicity (ADCC) (13, 47–49). Prior studies showed signs of transient grade 3 CRS (13). Current studies were designed to anticipate this possibility, as CRS was considered an on-target effect reflecting target engagement and the activation of an immune response (49). Furthermore, the oncology community has become more familiar with CRS management, and guidelines for management have been published (50). An ongoing phase 1 dose-escalation study in patients with advanced, refractory solid tumors is assessing CI-8993 (NCT04475523). Based on data published to date, the safety profile appears to be manageable, with no dose-limiting side effects observed up to the 0.6 mg/kg dose level (51). Pharmacokinetic analyses indicate that CI-8993 is demonstrating saturation binding kinetics to VISTA (51). Examination of the pharmacodynamic effects of CI-8993 has revealed increased maturation of NK cells, decreased recruitment of MDSCs, increased release of T-cell activating factors, and enhanced presentation of antigens on monocytes, suggesting that treatment may activate multiple anti-cancer mechanisms (51).

The complexity of targeting a relatively abundant protein such as VISTA with therapeutics that have a very strong binding affinity (Kd in the 350–500 picomolar range) leads to the phenomenon of target-mediated drug disposition (TMDD) (52). In TMDD, an abundant protein (such as VISTA) serves as a sink for the therapeutic agent, which then impacts the pharmacokinetics and therapeutic localization of the agent. Dr. Lionel D. Lewis of the Geisel School of Medicine at Dartmouth & The Dartmouth Cancer Center reviewed the properties of therapeutic agents (including mAbs) that undergo TMDD. Dr. Lewis emphasized the nonlinear and time-dependent change in the pharmacokinetics of such therapeutic agents and how there is a failure to saturate binding to VISTA, especially at lower doses, which in the setting of a high-affinity binding therapeutic can make the determination of optimal schedule and dosing very challenging. This phenomenon has been modeled across multiple therapeutics, reviewed by Dua et al. and Dostalek et al. (53, 54). Preclinical studies demonstrated bioavailability of a subcutaneously injected mAb that was inversely related to the dose level, likely due to saturable binding (55). Dr. Andrew M. Scott of the Olivia Newton-John Cancer Research Institute presented new data in a trio of tumorigenic mouse models using radiolabeled CI-8993 in conjunction with positron emission tomography imaging to demonstrate specific localization of CI-8993 to tumor sites, with evidence of VISTA receptor saturation at high doses. However, the amount of peripheral VISTA saturation needed to achieve the optimal antitumor effect dose in humans is pending further study.

A second anti-VISTA antibody, HMBD-002 (Hummingbird Bioscience), is also being investigated in clinical trials. HMBD-002 was rationally designed to bind to VISTA by targeting key residues within the C-C’ loop predicted to be involved in mediating interactions with VISTA binding partners (13, 47). In this case, the antibody is designed with an IgG4 Fc, which does not activate ADCC, allowing the preservation of important VISTA+ immune cells while stimulating the desired proinflammatory cytokine response from VISTA blockade (13). HMBD-002 has demonstrated significant inhibition of tumor growth in multiple solid tumor models, accompanied by increased numbers of antigen-presenting cells and reduced numbers of MDSCs in the TME (13). Additional preclinical validation was demonstrated by enhanced activation of CD8+ T cells and proinflammatory reprogramming of TAMs (56). Responses to HMBD-002 have also been evaluated in combination with pembrolizumab in a colon cancer model, demonstrating a synergistic antitumor effect (56). HMBD-002 is currently being assessed in an ongoing phase 1/2 dose-escalation trial (NCT05082610), which will include a combination arm with pembrolizumab. Thus far, HMBD-002 has shown a promising profile and has been safely tolerated at doses of up to 180 mg weekly (57).

The anti-VISTA antibody KVA12123 (Kineta, Inc.) is a fully human-engineered IgG1 mAb designed to bind to VISTA through a unique epitope in the extracellular domain, blocking interactions with putative VISTA binding partners, and blocking VISTA function (58). It has been engineered to provide strong single-agent tumor growth inhibition with no demonstrated CRS-associated cytokine release observed in preclinical models (59). Additionally, the effects of KVA12123 have been shown to reverse immunosuppression in the TME by increasing infiltrating T effector cells (CD4+ and CD8+), increasing the ratio of M1 macrophages, and enhancing the activation of NK cells (60). Tumor growth inhibition has been observed with KVA12123 treatment in multiple preclinical models, including colon, bladder, and T-cell lymphoma, as a monotherapy and in combination with PD-1 inhibitors (60). Studies in non-human primates have shown KVA12123 to be well tolerated at doses up to 100 mg/kg (60). A phase 1/2 open-label trial of KVA12123 alone and in combination with pembrolizumab in patients with advanced solid tumors (NCT05708950) has been initiated with planned increasing dose cohorts of KVA12123 from 3 mg up to 1000 mg.

Determination of the crystal structure of the extracellular domain of VISTA has provided insights into binding of both putative VISTA ligands and VISTA antibodies (47). This includes an extended C-C’ loop, which contributes to epitopes for multiple VISTA antibodies, including the previously described CI-8993 and HMBD-002 (13, 47). CI-8993 and two preclinical anti-VISTA antibodies, BMS767 and SG7, have been reported to have overlapping epitopes but different crucial amino acids for VISTA binding (13, 48). The three antibodies have been demonstrated to block both each other and interactions with human PSGL-1 and VSIG-3, with BMS767 doing so in a pH-dependent manner, reviewed elsewhere (48).

## Conclusions and future directions

Research shows that the VISTA checkpoint is differentiated from similar molecules with important roles in the regulation of myeloid cells and the promotion of immune system quiescence. Presentations at the VISTA symposium further demonstrated that anti-VISTA treatment might show synergy with anti-PD-1 and anti-CTLA-4 agents due to a reduction in the suppressive character of myeloid cells in the TME and complementary pro-activation effects on T cells. These findings suggest that anti-VISTA treatment may help to overcome the emergence of adaptive resistance to common ICI therapies, and combinations of these approaches will be explored in clinical trials. Antibodies targeting VISTA entering the clinic are beginning to yield promising outcomes; however, more assessment and development are needed to understand the clinical consequences of different antibody Fc isotypes and to manage treatment-related adverse effects like CRS. Beyond its role in the regulation of tumor immunity, VISTA has been shown to play a central role in the development of immunity and autoimmune disease through its negative regulatory role in controlling the innate and adaptive arms of the immune response (5, 61–64).

Despite the progress in understanding VISTA, much remains to be learned. Future research must examine the physiological relevance of the proposed ligands for VISTA and under what conditions these interactions are most likely to occur. The functions of VISTA within the immune system must be further explored, both within the TME and in its roles in innate and adaptive immunity. The significance and prognostic relevance of VISTA expression in different tumor samples require further analysis to guide the selection of patients most likely to benefit. While VISTA expression measured by immunohistochemistry could be developed as a biomarker to guide patient selection for VISTA-targeted therapies, currently no standardized scoring method exists (3). Similar to that established for PD-L1, there is likely a need to assess the proportion of VISTA expression on tumor cells versus immune cells in the TME to properly guide patient selection.

Beyond the TME, there is an increasing evidence base indicating that gut microbiota-immune system interactions affect cancer progression and response to therapies (65). The efficacy of anti-PD-1-based immunotherapy has been associated with particular commensal microbial composition (66). Compositional deviations of gut microbiota have been correlated with both cancer prognosis and likelihood of response to specific ICIs (67). The composition of a patient’s gut microbiota may play a role in clinical response to anti-VISTA therapy and potentially could be a consideration in patient selection.

Further preclinical research, clinical trials, and discussions will be necessary to address the issues of specific tumor types most responsive to VISTA blockade, the effects of prior therapies, and potential biomarkers most predictive of clinical response, all of which are likely to substantially impact the beneficial effects of VISTA blockade in cancer patients.

## Author contributions

All authors listed have made a substantial, direct, and intellectual contribution to the work, and approved it for publication.
